# Effects of mindfulness training on perceived stress, self-compassion, and self-reflection of primary care physicians: a mixed-methods study

**DOI:** 10.3399/bjgpopen18X101621

**Published:** 2018-11-28

**Authors:** Herman van Wietmarschen, Bram Tjaden, Marja van Vliet, Marieke Battjes-Fries, Miek Jong

**Affiliations:** 1 Scientist, Department of Nutrition & Health, Louis Bolk Institute, Bunnik, Netherlands; 2 Trainer, Aandachtigedokters, Zeist, Netherlands; 3 Scientist, Department of Nutrition & Health, Louis Bolk Institute, Bunnik, The Netherlands; 4 Scientist, Department of Nutrition & Health, Louis Bolk Institute, Bunnik, The Netherlands; 5 Associate Professor, Department of Health Sciences, Mid Sweden University, Sundsvall, Sweden; 6 Scientist, Department of Nutrition & Health, Louis Bolk Institute, Bunnik, The Netherlands

**Keywords:** primary health care, general practice, general practitioners, mindfulness, stress, resilience

## Abstract

**Background:**

Primary care physicians are subjected to a high workload, which can lead to stress and a high incidence of burnout. A mindfulness training course was developed and implemented for primary care physicians to better cope with stress and improve job functioning.

**Aim:**

To gain insight into the effects of the mindfulness training on perceived stress, self-compassion, and self-reflection of primary care physicians.

**Design & setting:**

A pragmatic mixed-methods pre–post design in which physicians received 8 weeks of mindfulness training.

**Method:**

Participants completed validated questionnaires on perceived stress (Perceived Stress Scale [PSS]), self-compassion (Self-Compassion Scale [SCS]), and self-reflection (Groningen Reflection Ability Scale [GRAS]) before the training, directly after, and 6 months later. Semi-structured interviews were conducted with six participants after the training and a content analysis was performed to gain in depth understanding of experiences.

**Results:**

A total of 54 physicians participated in the study. PSS was reduced (mean difference [MD] -4.5, *P*<0.001), SCS improved (MD = 0.5, *P*<0.001), and GRAS improved (MD = 3.3, *P*<0.001), directly after the 8-week training compared with before training. Six months later, PSS was still reduced (MD = -2.9, *P* = 0.025) and SCS improved (MD = 0.7, *P*<0.001). GRAS did not remain significant (MD = 2.5, *P* = 0.120). Qualitative analysis revealed four themes: being more aware of their own feelings and thoughts; being better able to accept situations; experiencing more peacefulness; and having more openness to the self and others.

**Conclusion:**

Mindfulness training might be an effective approach for improving stress resilience, self-compassion, and self-reflection in primary care physicians.

## How this fits in

Physicians are subjected to a high workload leading to stress and burnout. Research is showing positive effects of mindfulness on stress and burnout-related symptoms. However, only few studies reported effects on validated stress and self-compassion scales 6 months post-intervention and none of those included a qualitative analysis of those effects. This study reports a reduction of perceived stress and an improvement of self-compassion up to 6 months after the training course. Qualitative analysis revealed the process of changes in self-reflection and perception of primary care physicians during those 6 months. Further pragmatic comparative studies are needed to better understand the benefits of mindfulness for primary care physicians.

## Introduction

*'The pressure, you create it yourself.'* (Interviewee 5)

Dutch primary care physicians report high levels of distress and burnout. According to a study by Twellaar *et al*,^1^ 20% of Dutch primary care physicians suffered from burnout. This appears to be an international issue, as other countries report similar high burnout prevalence rates.^2^ Burnout is the unintended result of disruptive changes in the society, the medical profession, and the healthcare sector.^3^ As a result of government policies, which aim to manage the effects of the general ageing of the population on healthcare costs, more tasks are being diverted from hospital care to primary care. This results in an increase of the already high workload of primary care physicians. Furthermore, physicians are commonly trained to take care of the patient first, often making heroic efforts and sacrifices to do so.^4^ However, this work attitude cannot be maintained without sufficient care for oneself. Prevention of burnout and discovering the processes underlying burnout are highly relevant today.

Within primary care, burnout has been defined as a syndrome characterised by emotional exhaustion, depersonalisation (experiencing patients as objects), and low sense of accomplishment.^5^ Verweij *et al* ^6^ state that burnout is not only a problem for primary care physicians themselves, but also for their patients. It can result in medical mistakes and a diminished quality of care.^7^

Mindfulness seems to be a promising intervention for reducing stress and enhancing wellbeing of physicians. Mindfulness was defined by Kabat-Zinn as paying attention in the here and now in a conscious and non-judgemental way. According to Shapiro *et al*,^8^ this definition embodies three axioms or building blocks of mindfulness: intention, attention, and attitude. These are interwoven aspects of a cyclic process and can be presented in a dynamic mindfulness model. Mindfulness brings about a shift in perspective that enables one to adopt a more detached attitude in life. From this, additional mechanisms may arise that lead to change and positive outcomes such as self-regulation, value clarification, and psychological flexibility.

Previous studies that investigated the effects of an educational programme for mindful communication in primary care physicians, reported that the programme was effective in terms of improvements of mindfulness, burnout, and empathy, at least up to 15 months after the programme. More recently, a pilot study was conducted using an adapted and abbreviated Mindfulness Based Stress Reduction (MBSR) course that demonstrated reduced stress and burnout in primary care physicians, 9 months after the course.^9^ Previously, a waiting-list controlled study was conducted in the Netherlands, examining the feasibility and short-term effectiveness of a MBSR programme for trainers (who were primary care physicians themselves)of primary care physicians associated with two Dutch training hospitals, using both quantitative and qualitative outcome measures.^6^ The MBSR programme appeared to be feasible and acceptable for the trainers. Compared with the control group, depersonalisation decreased, and dedication and mindfulness skills increased. The qualitative data indicated that participants became more aware and self-reflective. Increased wellbeing and a less judgemental and kinder attitude towards the self and others was also reported.^6^

The aim of the present study was to investigate the feasibility and effects of a mindfulness training course, primarily developed and implemented for primary care physicians to better cope with stress, in a more representative sample of primary care physicians in the Netherlands. Besides perceived stress, self-compassion was chosen as an outcome measure since it is an important target for reducing burnout in healthcare professionals and it is currently unknown whether a mindfulness intervention can enhance self-compassion.^10^ Self-reflection was chosen as another outcome measure to evaluate the effects of the mindfulness training. A pragmatic effectiveness mixed-methods design was chosen to investigate effects on perceived stress, self-compassion, and self-reflection directly after the training and 6 months post-intervention, as well as to gain more in-depth understanding of the experiences of participating in a mindfulness training course.

## Method

### Study design and participants

The present study was designed as a pragmatic pre–post effectiveness study evaluated by a mixed-methods approach.^11^ Physicians registering for the annually offered mindfulness training were provided with written information to inform them about the background and purpose of the study, mode of participation, and confidentiality of data. Informed consent was obtained from all participants before the start of the mindfulness training.

### Intervention

The mindfulness training was announced as an attention training for primary care physicians, based on the principles of mindfulness. The mindfulness training in this study was based on the MBSR training developed by Jon Kabat-Zinn,^12^ but central elements of the MBSR training, such as the seven pillars of mindfulness and the different meditations, were adapted for primary care physicians, to help them to incorporate these exercises in their professional life. For example, walking meditation was introduced in the context of walking to the waiting room of patients and back. The training consisted of weekly group sessions for 8 weeks, totalling 26 hours. Physicians participated in any one of the five mindfulness trainings that were organised during the period 2015–2016. The training was self-paid, at a cost of €750. The training included formal exercises (sitting meditation, body scan, mindful yoga, walking meditation) as well as informal exercises such as attention for daily tasks in work setting, especially with patients. Specific attention was given to the challenges that might occur in the implementation of mindfulness in the practices of primary care physicians. The mindfulness training was given by two qualified MBSR trainers, with ≥﻿5 years of experience in giving this training. The training was accredited by the Royal Dutch Medical Association.

### Quantitative analysis

#### Outcome measures

Outcome measures were changes in perceived stress, self-compassion, and self-reflection ability, directly after the training and 6 months later. The validated 10-point PSS developed by Cohen *et al* was used to measure perceived stress.^13^ Self-compassion was measured with the validated SCS developed by Neff *et al*.^14^ Personal ability to reflect was measured with the validated GRAS designed by Aukes *et al*.^15^ Additionally, self-report questionnaires before and after the training were used to collect demographic data of participants, and data was collected on relevance of the training and implementation of the training in daily and professional life of participants 6 months after the training. Questionnaires were provided and managed with the online service MWM2 .

#### Analysis

Descriptive statistics were performed on the demographic data. Paired student *t*-tests were performed to analyse the differences between pre-training and directly post-training, as well as between pre-training and 6 months after the training, after checking the variables for normality. Effect sizes were calculated (Cohen’s *d*) for the pre- and post-differences.^16^ All analysis of the data were performed with SPSS (version 24).

### Qualitative analysis

#### Data collection

Overall, sampling was based on the concept of ‘information power’. According to this model, sufficient information power depends on: (a) the aim of the study; (b) sample specificity; (c) use of established theory; (d) quality of dialogue; and (e) analysis strategy. The larger information power the sample holds, the lower number of participants is needed.^17^ As the qualitative interviews were additional to the quantitative study, and some qualitative data is available from a previous feasibility study about a MBSR training among Dutch physicians (data not published), a sample of six participants was considered appropriate. To increase sample specificity, the authorsaimed to include participants distributed over all age groups, both sexes, and from each of the offered courses.^18^ Participants were recruited on a voluntary basis and interviewed 3 months after the mindfulness training. As the level of comfort influences the quality of dialogue (and thereby the level of information power), in-depth interviews were conducted at a place chosen by the interviewee.^17,19^ Furthermore, interviews were conducted by the third author who had not been part of the training and has previous experience in interviews about mindfulness-based interventions among healthcare professionals. A topic guide was prepared to support the interview and interviewees were encouraged to tell stories and narratives about these topics in order for an understanding of their experiences to be obtained.^19^ Interviews were audio-recorded and transcribed for analysis.

#### Analysis

Content analysis was chosen owing to its flexibility and the exploratory character of the study.^20^ With this method, the authors aimed to provide a general account of the important and sensitive phenomena under study; that is, the common experiences of the participants with respect to the MBSR course.^21^ An inductive approach was chosen in which themes were tightly linked to the data, and not to pre-defined concepts.^22^ Data analysis were performed in line with the steps described by Elo and Kyngas.^21^ It consisted of first reading the transcripts and writing down impressions. Next, the data were open-coded by marking all meaningful expressions. Codes were then sorted based on their mutual content. Themes, subthemes, and corresponding names were formulated. Subsequently, themes and subthemes were checked by rereading the transcripts in the light of these themes. Themes and subthemes were reorganised and pieces of transcripts recoded. The process of rereading and recoding was repeated several times until a group of coherent themes and sub-themes emerged from the data. Analysis was mainly performed by the first author. To increase trustworthiness, the third author took the findings of each consecutive step under consideration and differences in opinion were discussed and agreed on.^23^

## Results

A total of 54 physicians participated in the study and completed the pre-training questionnaire. Fifty-one (94%) participants completed the questionnaire directly after the mindfulness training and 23 (43%) participants completed the questionnaire 6 months after the end of the training. Participants were reminded weekly, for up to 3 weeks, to complete the 6-month questionnaire.

Demographic characteristics of the 54 participants are presented in Table 1. All participants were physicians, the majority (87%) being primary care physicians. Most physicians were female (78%) with an average age of 40 years (Table 1). Of the 54 participants, 46 (85%) reported having some knowledge about mindfulness. One or more mindfulness and/or other mind–body practices were practised by 46 (85%) of the participants before or during the current mindfulness training. Twenty-two (41%) of the 54 participants had been practising some form of body–mind exercise within the 12 months before the start of the training: 10 (19%) less than monthly, seven (13%) monthly, and five (9%) weekly or daily.

**Table 1.** Demographic characteristics of participants (*n* = 54)

	*n* (%)
**Age, years (SD)**	40 (9)
**Sex**	
Male	12 (22)
Female	42 (78)
**Occupation**	
Primary care physician	47 (87)
Other	3 (6)
Not known	4 (7)
**Work setting**	
Own practice	5 (9)
Duo practice	8 (15)
Group practice	3 (6)
Healthcare centre	5 (9)
Observing physician	22 (41)
Other	11 (20)
**Years working as physician**	
<2 years	10 (19)
2–5 years	17 (31)
6–10 years	13 (24)
11–20 years	5 (9)
>20 years	9 (17)
**Experience with mindfulness**	
Not familiar	8 (15)
Familiar but never practised	24 (44)
Practises in the past or currently	22 (41)
**Types of practices (*n* = 22)**	
Mindfulness meditation	6 (27)
Other form of meditation	5 (23)
Yoga	15 (68)
Tai chi	4 (18)
Guided visualisation	3 (14)
Hypnotherapy	0 (0)
Relaxation exercises	11 (50)
Other	2 (9)
**Frequency of practices in past 12 months**	
Not	32 (59)
Less than once per month	10 (19)
Approximately monthly	7 (13)
Approximately weekly	1 (2)
Several times per week	2 (4)
Daily	2 (4)
Several times per day	0 (0)

### Quantitative results

The data on perceived stress, self-compassion, and self-reflection was normally distributed allowing t-tests to test for significant differences between groups. As shown in Table 2, after 8 weeks of mindfulness training, PSS was significantly reduced, SCS significantly improved, and GRAS significantly improved. Reduction in PSS and improvement of SCS were large effects, while the improvement on GRAS was a medium effect size (see Table 2).

**Table 2.** Differences in perceived stress, reflection, and self-compassion between 'before' and 'directly after' the mindfulness training

	*n*	Before, mean (SD)	Directly after, mean (SD)	Mean difference, (95% CI)	*P* value	Cohen’s *d*
Perceived stress (PSS)	49	17.9 (5.6)	13.4 (5.8)	-4.5 (-6.0 to -3.0)	0.000	-0.9
Self-compassion (SCS)	50	2.9 (0.7)	3.4 (0.6)	0.5 (0.3 to 0.6)	0.000	0.8
Reflection (GRAS)	44	87.6 (7.7)	90.9 (6.7)	3.3 (1.5 to 5.2)	0.001	0.5

CI = confidence intervals. GRAS = Groningen Reflection Ability Scale. PSS = Perceived Stress Scale. SCS = Self-Compassion Scale.

Six months later, PSS remained significantly reduced and SCS improved even more (Table 3). GRAS did not remain significant 6 months after the training. Reduction of PSS was a medium effect and the improvement of SCS a large effect size (Table 3).

**Table 3.** Differences in perceived stress, reflection and self-compassion between 'before' and '6 months after' the mindfulness training

	*n*	Before,Mean (SD)	6 months after,Mean (SD)	Mean difference, (95% CI)	*P* value	Cohen’s *d*
Perceived stress (PSS)	22	16.3 (6.1)	13.5 (6.3)	-2.9 (-5.4 to -0.4)	0.025	-0.5
Self-compassion (SCS)	21	2.9 (0.7)	3.7 (0.7)	0.7 (0.4 to 1.0)	0.000	1.2
Reflection (GRAS)	17	87.7 (9.8)	90.2 (10.9)	2.5 (-0.7 to 5.7)	0.120	0.4

CI = confidence intervals. GRAS = Groningen Reflection Ability Scale. PSS = Perceived Stress Scale. SCS = Self-Compassion Scale.

Participants agreed to a high extent that the mindfulness training was useful for primary care physicians (4.5 ± 0.8; on a scale from 1 [totally disagree] to 5 [totally agree], and contributed to stress-reduction (4.5 ± 0.8) and to the professional development of primary care physicians (4.4 ± 0.6). Furthermore, 6 months after the training, a substantial percentage of physicians still practised exercises as taught in the mindfulness training such as yoga (45%), 3-minute meditation (45%), walking meditation (35%), and the body scan (30%).

### Qualitative results

Four themes and eight subthemes were extracted from the interview data (see Box 1).

**Box 1.** Overview of the themes and subthemes


**Themes**	Self-awareness	Acceptance	Peacefulness	Openness
**Sub-themes**	Awareness of feelings, thoughts and behaviour patterns	Self-acceptance	Stress relief	Perspective taking
	Discovering boundaries	Re-evaluation of situation	Achieving mindfulness	Openness towards other approaches

#### Self-awareness

##### Awareness of feelings, thoughts and behaviour patterns

The first subtheme was about the effect of the mindfulness training on increasing awareness of feelings, thoughts, and behaviour patterns. Physicians were more able to stop for a moment and think about what they were doing. This allowed them to better perceive thoughts that contributed to stress:

*‘* [...] *you sometimes think for a moment: yeah, how does that influence my thoughts about the world. And to be aware of that, that I am judging things in this way, and that I am striving and not having a fresh look at things. You know, that is so nice to understand.’* (Interviewee 5)

##### Discovering boundaries

Physicians were better able to feel boundaries for themselves and towards patients; for instance, the ability to stay with yourself in dealing with difficult patients:

*‘* […] *there was a bit of gaining awareness of the consultation and then thinking, "Yes, I have become better in it." Because I dare to take a little distance now. Because this responsibility for her belly ache, I should be able to give that back to her. Yes, then I realise that I did take some steps.’* (Interviewee 4)

#### Acceptance

##### Self-acceptance

This subtheme concerned the increased self-acceptance, self-compassion, and gentleness towards the self that was experienced by the physicians:

*‘I am very strict with myself. Quite the perfectionist. And there has been a lot of attention to that during the training. Having compassion for yourself. And I am able to do that better and better.’* (Interviewee 1)

##### Re-evaluation of situations

Physicians also mentioned having a more neutral perspective on situations, and the ability to let situations be as they are:

*‘You pause for a bit and think: what is happening? Is it a tiger or is it just what it is? Do we have to do something with it or can it wait until later?’* (Interviewee 3)

#### Peacefulness

##### Stress relief

Physicians acquired tools for achieving relaxation such as building in brief moments to breathe and relax:

*‘Once in a while I take a small break to breath … Then I notice stress going down … So literally just notice what is happening in your body with your breathing and in your head at that moment.’* (Interviewee 3)

##### Achieving mindfulness

Physicians were able to achieve moments of mindfulness, such as consciously enjoying the moment and letting go of thoughts:

*‘Yes, yes, especially looking around me: to see what a nice colours or what a nice flowers and just really look at that for a moment.’* (Interviewee 2)

#### Openness

##### Perspective taking

Physicians reported increasing their understanding of others and being less judgemental about others because of the training:

*‘You realise a little quicker: why does someone think like that, or you can see it from another perspective. What was going on? Why does a patient want this, why does a colleague want this in a certain manner? You don’t always understand it very well and you can just be a little more open towards it.’* (Interviewee 2)

##### Openness towards other approaches

Physicians learnt to see other possible approaches to deal with certain situations with their patients:

*‘If they keep returning with the same complaints, what is behind it? Am I not able to help them in another way? Or deal with it in another manner. Addressing the mental side some more. Put more time in it.’* (Interviewee 1)

## Discussion

### Summary

This mixed-methods study demonstrated that participation in mindfulness training had beneficial effects for primary care physicians. The 8 weeks of mindfulness training significantly decreased perceived stress and improved self-compassion in primary care physicians up to 6 months post-intervention. Self-reflection was significantly improved directly after the training, but not at 6 months after the training. Furthermore, the qualitative data revealed that participation in the mindfulness training made the participants more aware of their own feelings and thoughts, and better able to accept situations, experience more peacefulness, and have more openness to the self and others.

### Strengths and limitations

A strength of the present study was the use of mixed methods; the magnitude of effects on the validated scales of perceived stress, self-compassion, and self-reflection were complemented by the qualitative findings about the experiences of the MBSR course among the participants. Another strength was the 6-month post-intervention follow-up of the study, demonstrating that effects were sustained over half a year. This might be related to the finding that a substantial number of participants had been able to implement mindfulness exercises into their daily life after the training. The study had several limitations as well. First of all, the study was not of a randomised design and lacked an appropriate control group, such as a wait-list control group. It was deemed unethical to establish a wait-list or other control group for this study, since physicians registering for the mindfulness training wanted to start directly with the training and payed for it themselves. Furthermore, it was not quantitatively measured whether the observed beneficial changes were associated with higher levels of mindfulness in the participating physicians after the training. However, the qualitative findings in the present study suggest so, as more awareness, acceptance, and openness were perceived by physicians as a result of the mindfulness training. Another limitation of the study was that participating physicians were self-selected, and thus the sample might have included those who experienced more stress, or had a specific interest in mindfulness. Generalising the findings to other primary care physicians should, therefore, be done with caution. Another limitation was the percentage loss to follow-up with respect to the response rates at 6 months after the training. This carries the risk that participants who experienced more beneficial effects from the course were more likely to respond than those with negative experiences. However, there were no significant differences in perceived stress, self-compassion, and self-reflection between the 6-month responders and non-responders at baseline and at 3 months. Furthermore, the experiences of those subjects with types of mind–body exercises as well as the frequency of practice 12 months before the training were also similar between the 6-month responders and non-responders, thereby, reducing concerns of a response bias. An additional limitation is the voluntary participation of the physicians for the interviews, which may have resulted in a selection of those with a more general positive attitude towards the mindfulness training. However, specific attention to both positive and negative experiences of the participants was addressed in the interviews.

### Comparison with existing literature

The findings in the present study are well in line with previously published studies of different designs, and add to the growing evidence for the effectiveness of mindfulness interventions in reducing burnout-related symptoms and improving wellbeing in primary care physicians.^6,9,24^ A previous study of Dutch trainers of GPs reported on qualitative findings that the mindfulness intervention increased self-compassion. Importantly, this result was confirmed quantitatively in the present study, using the validated self-compassion scale of Neff *et al*.^14^ It has been demonstrated that the negative dimensions of self-compassion, such as self-judgement, isolation, and over-identification, are underlying factors that can explain the vulnerability of certain primary care physicians to develop burnout.^25^ It seems, therefore, of utmost importance to provide primary care physicians with techniques for training self-compassion, by means of a mindfulness training, such as described in the present study, in order to prevent burnout and poor wellbeing.

### Implications for research and practice

It is evident from the literature that a substantial percentage of primary care physicians experience high levels of chronic stress in response to workforce and workload issues, eventually leading to burnout.^1,2^ Burnout does not only affect the wellbeing of physicians themselves, but also can have a negative impact on patients. It has been demonstrated that the level of burnout in healthcare professionals is associated with poorer quality of patient care, related to decreased empathy towards the patient, more medical errors, and lower patient satisfaction.^26–28^ The findings of the present study suggest that providing a mindfulness training course to primary care physicians, outside their workplace, is recommended to prevent burnout, improve their wellbeing, and consequently improve patient care. In terms of burnout prevention, however, it might seem more effective to start as early as possible by providing the mindfulness training in the medical curriculum as for medical students to learn how to better cope with stress in future situations. Introduction of mind–body techniques, such as mindfulness into the medical curriculum, has been shown to reduce stress and increase empathic concern in medical students.^29–31^ A recently published study provided preliminary evidence that the beneficial effects of such training last until after graduation, and resulted in better self-care and better patient care.^32^ Participants who were interviewed in the present study recommended implementing the mindfulness training in the regular required medical curriculum, but had different views on when to provide the training in the curriculum. Further research is needed to investigate whether the training should be delivered to medical students at a certain point in the medical curriculum for optimum effectiveness;^31^ for example, right before they enter clinical practice, when they start to see patients.

The mindfulness training, as described in the present study, was specifically developed for primary care physicians and therefore comprises an intervention that is directed at them. In order to prevent work-related stress and burnout, it seems relevant to also change the working environment of primary care physicians. A focus group study explored strategies that could improve wellbeing of primary care physicians. This study identified compulsory daily breaks, (peer) support systems, more staff, and more time for patient contacts as possible effective strategies.^33^ However, empirical evidence for organisation-directed interventions, such as reducing the working hours, having peer-support groups, or improving communication skills of healthcare professionals, is limited.^34^ Furthermore, primary care physicians may have few opportunities to make structural changes in their working environment, owing to the many tasks and challenges they are faced with and the way the primary healthcare system is organised.^35–36^ Thus, if the healthcare system cannot be changed so easily, it is important to support self-care of physicians. Self-care is defined as 'a cadre of activities performed independently by an individual to promote and maintain personal wellbeing throughout life'.^37^ Developing self-awareness and self-reflection seem to be key factors in self-care for physicians.^37–38^ Since the present study demonstrated that a mindfulness training can enhance self-awareness of physicians, it is recommended that primary care physicians include such training in their self-care planning. Little is known about which elements in the mindfulness training may positively contribute to reduce stress, increase self-awareness, and increase self-compassion in physicians. This might also depend on individual preferences,^39^ burnout subtypes,^25^ and the working environment of primary care physicians. Further pragmatic research is, therefore, warranted that tailors the mindfulness training to individual and organisational needs in order to maximise its effectiveness.
